# Supplementary material for: Dilemma of physician-mothers faced with an increased home burden and clinical duties in the hospital during the COVID-19 pandemic
Source: PLoS One. 2021 Jun 24;16(6):e0253646. doi: 10.1371/journal.pone.0253646 (PMC8224842; doi:10.1371/journal.pone.0253646)
Supplement: S1 File — (DOCX) [file pone.0253646.s002.docx]

**S1 File.**

**Q1. General Questions**

**Q1-1. Age (years)**

□ 20～29 □ 30～39 □ 40～49 □ 50～59 □ 60～69

**Q1-2. Gender**

□ Man □ Woman □ Other, including not willing to answer this question

**Q1-3. Employment position**

□ Attending Physician □ Clinical fellow □ Resident □ Researcher* □ Graduate student**

*, ** with Medical Doctor license, finished residency and engaged in treatment/care of COVID-19 patients

**Q2. Experience of treating/caring for patients with COVID-19 in Sapporo Medical University Hospital during the 1st and 2^nd^ waves**

□ Yes □ No

**Q3. Experience of treating/caring for patients with COVID-19 in other hospitals during the 1st and 2^nd^ waves.**

□ Yes □ No

**Q4-1.** **Sapporo Medical University Hospital accepted many COVID-19 patients, as reported in the press. Did you experience any discrimination or prejudice as a result?**

**Q4-2．If yes in Q4-1, please describe the details (free comment, optional).**

**Q5-1. Has there been any change in your household burden during the 1st and 2^nd^ waves?**

□ No, not increased/equivocal

□ Yes, slightly increased

□ Yes, markedly increased

**Q5-2．If you checked Yes in Q5-1,** **slightly/markedly increased, please describe in detail (multiple choice, optional).**

□ temporarily increased housework regarding childcare, associated with the closure of childcare service or school due to the pandemic

□ learning support in home

□ mental support in home

□ housekeeping and/or cooking

□ closure of a facility for elderly family member(s)

□ others (free comment)

**Q6．Do you have any dependent relatives? If you checked Yes, please select all that apply.**

□ No

□ Yes

□ children

□ adult children

□ pregnant

□ elderly

**Q7．If you have a child, who mainly performed childcare in your home during the 1^st^ and 2^nd^ waves of the pandemic?**

□ mainly myself

□ mainly my partner

□ grandparents

□ equivocal/other

**Q8. Wage earner in your family**

□ two income

□ myself or partner

□ other

**Q9. Free comment**
