# Supplementary material for: Dilemma of physician-mothers faced with an increased home burden and clinical duties in the hospital during the COVID-19 pandemic
Source: PLoS One. 2021 Jun 24;16(6):e0253646. doi: 10.1371/journal.pone.0253646 (PMC8224842; doi:10.1371/journal.pone.0253646)
Supplement: Attachment — Submitted filename: Review Comments to the Author.docx [file pone.0253646.s003.docx]

4. Is the manuscript presented in an intelligible fashion and written in standard English?

Word had better change:

domestic burden → burden of household chores

double-income → two-income

attending physicians → physicians

social peoples → citizens

5. Review Comments to the Author

1) I've pointed out some words that should be changed so check it out.

2) **Abstract**

Purpose: The purpose of this study was to evaluate whether the burdens are overly concentrated on physicians, especially female doctors, in our hospital. →

The purpose of this study was to assess how much the burden on our doctors, especially female doctors, was increasing.

Conclusion: This suggests that we have to redress this inequality as soon as possible to avoid such problems in the event of another such social crisis, which could occur at any time. →

In order for female doctors to continue their careers and take responsible positions in the same way as male doctors, it is necessary to improve the delay in gender equality in Japanese society.

3) **Introduction**

Importantly, during the pandemic, physicians in our hospital had to respond both to the issue of treating patients and ~~the increased domestic burden~~ spending more time on house hold activities associated with social disruption. The current COVID-19 pandemic has been described to intensify workplace inequities for women physician[1], in this situation, there is especial concern that the ~~domestic~~ burden of housework can be over-concentrated on female physicians and create a dilemma when faced with both internal and external duties. ~~will be over-concentrated on female physicians and could cause a dilemma when they are faced with duties both in and outside of the home.~~

For this reason, the Physicians’ Career Support Committee in Sapporo Medical University performed a questionnaire survey of physicians working in our university to assess the domestic burdens of the genders during the pandemic. From the results of the questionnaire, we analyzed the burden of housework, including the care of children mainly by gender, and examined problems related to women's career continuity.

4) **Results**

**Increase of domestic burden associated with the pandemic according to gender**

During the first and second waves of the COVID-19 pandemic, 62/264 (23.5%) and 23/264 (8.7%) answered that the domestic burden was slightly or markedly increased, respectively (Table 2). The increase was found for both genders. ~~However, regarding a marked increase of the domestic burden associated with the pandemic, the ratio of female physicians with such an increase was significantly higher than that of male physicians (not or equivocal/slight/marked increase; 127 (71.3%)/39 (21.8%)/11 (6.2%) for male physicians vs. 49 (59.8%)/21 (25.6%)/12 (14.1%), respectively, for female physicians, p=0.04).~~ 28.1% (50/178) of men and 40.2% (33/82) of women answered that the burden of housework increased slightly or significantly, which was the significant burden for female physicians (p=0.04).

5) **Discussion**

Such impact has not been the same ~~across the all social peoples~~ among all citizens,

~~Before the COVID-19 pandemic, it was reported that female physicians are more likely to have partners who are employed full-time and report spending more time on household activities including cleaning, cooking, and the care of children, compared with their male counterparts[5]~~ According to a survey of household chores conducted by the Japan Surgical Society prior to the COVID-19 pandemic, male surgeons spent the same amount of time doing household chores with and without children. Female surgeons, with or without children, spent more time doing household chores than male surgeons [5]. ~~and some~~ Other studies have reported ~~showed that~~ on female physicians have struggle to balance their career and families. [6-8] Thus, the results showed that a significantly higher proportion of physician-mothers were caught in a dilemma between an increased home burden and clinical duties in the hospital than physician-fathers during the pandemic. The results showed that during the pandemic, physician-mothers showed more difficulty balancing career and increased household chores than physician-fathers.

In the first and second waves in Japan, schools and nurseries were closed by the government, resulting in more children having to be cared for at home　~~an increase in　the number of children who stayed at home.~~

~~This might be because some young female physicians might have left their careers as physicians to parent children.~~ From this result, it can be inferred that it is difficult for female doctors to continue working when the number of dependents increases, and it is difficult for female doctors to work in hospitals for a long period of time until they take a responsible position. These tendencies were not only typically shown in our hospital but in the entire Japanese health care system. ~~In other words, such differences might be related to delayed systems of childcare in Japan.~~ This is because Japanese society does not have a sufficient environment for women to continue their careers. Actually, according to the Global Gender Gap Report 2021[11] , Japan ranks 120th out of 156 countries in the comprehensive index of gender equality of health, education, economy, and politics, indicating that the social progress of females in this country lags far behind other nations.

6) **Conclusions**

Our results show that a significantly higher proportion of physician-mothers were caught in a dilemma facing an increased home burden and clinical duties in the　hospital than physician-fathers during the pandemic. ~~This suggests that we have to redress this inequality as soon as possible to avoid such problems in the event of another such social crisis, which could occur at any time.~~ In order for female doctors to continue their careers and take responsible positions in the same way as male doctors, it is necessary to improve the delay in gender equality in Japanese society.

7)  **References**

 4. The impact of COVID-19 on women [Internet]. United Nations. 2020 [cited 2020 April 9]. Available from:  ~~https://www.un.org/sites/un2.un.org/files/policy_brief_on_covid_impact_on_women _9_april_2020.pdf.~~

<https://www.unwomen.org/en/digital-library/publications/2020/04/policy-brief-the-impact-of-covid-19-on-women>

or

https://www.unwomen.org/-/media/headquarters/attachments/sections/library/publications/2020/policy-brief-the-impact-of-covid-19-on-women-en.pdf

5. ~~Starmer AJ, Frintner MP, Matos K, Somberg C, Freed G, Byrne BJ. Gender Discrepancies Related to Pediatrician Work-Life Balance and Household Responsibilities. Pediatrics. 2019;144(4). Epub 2019/09/10. doi: 10.1542/peds.2018-2926. PubMed PMID: 31506304.~~

Kawase, K., Nomura, K., Tominaga, R., Iwase, H., Ogawa, T., Shibasaki, I., Shimada, M., Taguchi, T., Takeshita, E., Tomizawa, Y., Nomura, S., Hanazaki, K., Hanashi, T., Yamashita, H., Kokudo, N., et al. (2018) Analysis of gender- based di erences among surgeons in Japan: results of a survey conducted by the Japan Surgical Society. Part. 2: personal life. *Surg. Today*, **48**, 308-319.

11. ~~Health at a glance 2019 [Internet]. OECD publisher. 2019 [cited 2019 November 7]. Available from: https://doi.org/10.1787/4dd50c09-en.~~

The Global Gender Gap Report 2021: http://www3.weforum.org/docs/WEF_GGGR_2021.pdf
