# Supplementary material for: Dilemma of physician-mothers faced with an increased home burden and clinical duties in the hospital during the COVID-19 pandemic
Source: PLoS One. 2021 Jun 24;16(6):e0253646. doi: 10.1371/journal.pone.0253646 (PMC8224842; doi:10.1371/journal.pone.0253646)
Supplement: Attachment — Submitted filename: PONE response to reviewer_ver05.docx [file pone.0253646.s004.docx]

**Response to the comments by the reviewer**

Reviewer #1

A) Major
1) *Please mention the gender distribution of all 622 medical and dental physicians and researchers working in Sapporo Medical University.*

Thank you for pointing that out. At the time of survey, there were 459 male and 163 female doctors in Sapporo Medical University. We added the following sentence with underline.

The site information (the URL) of the questionnaire was announced to all 622 medical and dental physicians and researchers, including 459 males and 163 females, working in Sapporo Medical University by e-mail on September 8, 2020.

2) *The general background in your institution is well written in Introduction. However, please mention what kind of physician take care for COVID-19, how to share the work, and the role of researcher in your institution.*

Thank you pointing that out. We add the following sentences with underline in introduction.

As well as clinical physicians, basic researchers and graduate students with medical doctor license were involved in the diagnosis of the COVID-19 suspected cases in the rural hospitals, too.

3) *Please mention regarding the social support system in Japan in Discussion.*

Thank you pointing that out. We added the following paragraph about the social support for the childcare:

In Japan, most of the social support for a female doctor under the COVID-19 pandemic was limited. One reason why such limitation was coursed will be a character of this social crisis and unbalanced social structure in Japan. The lock-down for the control of the pandemic stopped traffics between the city and rural areas. Thus, if one parent treated COVID-19 patients in the rural hospital and stayed there, the other had to care for their child in their urban residence. In the Japanese social structure, especially in Hokkaido, most of the population (approximately 40%) is focused on the city. For these reasons, the need for babysitting was markedly elevated in the city area, and the Japanese government introduced economic support policies for babysitting services. However, a conventional babysitter has a risk of becoming an infection transmitter. The person who had both experiences of caring for children and prevention of infection is less, comparing to the need. Regarding parenting children under pandemic, this issue cannot easily find the answer and is a common topic globally. Under this context, we want to emphasize the contradiction that, although female doctors cannot take responsible positions in pre-pandemic society, they are asked to be responsible for this challenging issue. According to our survey, most female physicians, not males, had difficulty finding the ideal babysitter and caring for children under this pandemic.

4) *You have to provide proof number of ethical approval in the institution even though the board chairman’s judgement in your institution.*

Thank you pointing that out. In the initial process regarding our application to the institutional review board and ethics committee of Sapporo Medical University, the board chairman judged that this study was not needed to review by a committee. But, in response to your suggestion, we applied for and received the approval. We added the following sentences.:

However, just in case, ethical approval for this study was obtained from the institutional review board and ethics committee of Sapporo Medical University (3-1-14).

Reviewer #2

1) *It is easier to use treating Covid-19 patient in the abstract.*

Thank you for the improvements. The following corrections have been made.

L 60 treating patients (p=0.03) were→　treating COVID-19 patients (p=0.03) were

2) *In Table. 4, was there an investigation into the changes in those responsible for caring for children before and after the pandemic?*

Thank you for your advice. In table 4, we asked the key person of childcare under the COVID-19 pandemic, so we did not examine the changes from the previous situation. We will continue to investigate in that regard as well.

3) *Of the 83 people who said they had to spent times for more housework, how many doctors had children? I think the impact of daycare centers and school closures is significant, but is there a link between children's age and the burden of housework?*

Of the 83 respondents who answered that their home burden had increased, 72 (84.7%) had children. On the other hand, of the 178 respondents who answered that the burden at home had not increased, 82 (46.0%) had children. This is the reason for the statistically significant difference in Table3. Meanwhile among the 18 respondents whose youngest child was a graduate student or working adult, 1 (5.0%) answered that the burden had increased significantly, 4 (22.2%) answered that it had increased slightly, and 13 (72.2%) answered that it had remained the same. There was no apparent difference from the overall results. The age of the child did not affect the increase in the burden on the family. So we added the following sentences with underline in the limitations:

Fourth, the rate of the increase of home burdens among doctors with adult children was not statistically different from those with young children, and we have not been able to investigate the causes of this increase due to adult children. It was thought necessary to look more deeply into whether adult children live with parents, or they are financially independent.

4) *Line 252: treating patient to treating COVID-19 patient*

Thank you for the improvement. I've fixed it as you pointed out.

Reviewer #3

*1)Word had better change:*

*1. domestic burden → burden of household chores*

*2. double-income → two-income*

*3. attending physicians → physicians*

*4. social peoples → citizens*

Thank you for pointing this out. I have corrected 2 and 4, except for the word of “domestic burden” and “attending physician”.

A) According to the Oxford English Dictionary, “chore” means a task that you do regularly and an unpleasant or boring task. Especially under the social crisis, there were many highly necessary tasks, including home hygiene and unexpected childcare, which does not mean just a routine task, so we want to use the word “domestic burden”. I hope you will agree.
B) In this paper, attending physician include assistant professor, instructor, associate professor and professor. The word of “faculty” may be better to understand for readers, but due to the characteristics of this study, we want to express them as clinicians. So, we use the word of “attending physician”. I hope your cooperation.

*2) Sentences that should be revised*

1. *Abstract Purpose: The purpose of this study was to evaluate whether the burdens are overly concentrated on physicians, especially female doctors, in our hospital. →*

*The purpose of this study was to* ***assess how much the burden on our doctors, especially female doctors, was increasing.***

Thank you for pointing this out. I have corrected it.

2. *Abstract Conclusion: This suggests that we have to redress this inequality as soon as possible to avoid such problems in the event of another such social crisis, which could occur at any time. →* ***In order for female doctors to continue their careers and take responsible positions in the same way as male doctors, it is necessary to improve the delay in gender equality in Japanese society****.*

As you pointed out, we believe that it is important to eventually raise the percentage of women in leadership positions. Although this study found that women were burdened, it did not prove directly that female doctors gave up their careers because of this burden. So, we revised as follows:

As we showed, female doctors could have not continued their careers and take responsible positions in the same way as male doctors. This is a social risk in the timing of a crisis, such as a pandemic.

3. *Introduction:*

*Importantly, during the pandemic, physicians in our hospital had to respond both to the issue of treating patients and ~~the increased domestic burden~~* ***spending more time on household activities*** *associated with social disruption. The current COVID-19 pandemic has been described to intensify workplace inequities for women physician[1], in this situation, there is especial concern that* ***the ~~domestic~~ burden of housework can be over-concentrated on female physicians and create a dilemma when faced with both internal and external duties.*** *~~will be over-concentrated on female physicians and could cause a dilemma when they are faced with duties both in and outside of the home.~~*

Thank you for pointing this out. I corrected as you suggested.

4. *Introduction:*

*For this reason, the Physicians’ Career Support Committee in Sapporo Medical University performed a questionnaire survey of physicians working in our university to assess the domestic burdens of the genders during the pandemic.* ***From the results of the questionnaire, we analyzed the burden of housework, including the care of children mainly by gender, and examined problems related to women's career continuity.***

Thank you for pointing this out. However, we are sorry, only in this study we did not look at women's career continuity under the pandemic. So, we modified the sentences as follows:

For this reason, the Physicians’ Career Support Committee in Sapporo Medical University performed a questionnaire survey of physicians working in our university to assess the domestic burdens of the genders during the pandemic. From the results of the questionnaire, we analyzed the burden of housework, including the care of children mainly by gender.

5. *Results: Increase of domestic burden associated with the pandemic according to gender*

*During the first and second waves of the COVID-19 pandemic, 62/264 (23.5%) and 23/264 (8.7%) answered that the domestic burden was slightly or markedly increased, respectively (Table 2). The increase was found for both genders. ~~However, regarding a marked increase of the domestic burden associated with the pandemic, the ratio of female physicians with such an increase was significantly higher than that of male physicians (not or equivocal/slight/marked increase; 127 (71.3%)/39 (21.8%)/11 (6.2%) for male physicians vs. 49 (59.8%)/21 (25.6%)/12 (14.1%), respectively, for female physicians, p=0.04).~~* ***28.1% (50/178) of men and 40.2% (33/82) of women answered that the burden of housework increased slightly or significantly, which was the significant burden for female physicians (p=0.04).***

Thank you for pointing this out. Statistically, there is a significant difference between the groups responded the burden of housework increased markedly. I apologize for the misunderstanding. The details are shown in the shaded area, so we will submit the original sentences.

6. *Discussion*

*Such impact has not been the same ~~across the all social peoples~~* ***among all citizens****,*

Thank you for pointing this out. I corrected the sentences.

7. *~~Before the COVID-19 pandemic, it was reported that female physicians are more likely to have partners who are employed full-time and report spending more time on household activities including cleaning, cooking, and the care of children, compared with their male counterparts[5]~~* ***According to a survey of household chores conducted by the Japan Surgical Society prior to the COVID-19 pandemic, male surgeons spent the same amount of time doing household chores with and without children. Female surgeons, with or without children, spent more time doing household chores than male surgeons [5]. Other studies have reported*** *~~showed that~~ on female physicians have struggle to balance their career and families. [6-8]*

Thank you for your especially important point. I have cited the paper you pointed out.

8. *Thus, the results showed that a significantly higher proportion of physician-mothers were caught in a dilemma between an increased home burden and clinical duties in the hospital than physician-fathers during the pandemic.　→* ***The results showed that during the pandemic, physician-mothers showed more difficulty balancing career and increased household chores than physician-fathers.***

Thank you for pointing this out. We believe that it's not all boring chores that have increased during the pandemic. So, we want to use original sentence.

9. *In the first and second waves in Japan, schools and nurseries were closed by the government,* ***resulting in more children having to be cared for at home***　*~~an increase in　the number of children who stayed at home.~~*

Thank you for pointing this out. I corrected as you suggested.

10. *~~This might be because some young female physicians might have left their careers as physicians to parent children.~~* ***From this result, it can be inferred that it is difficult for female doctors to continue working when the number of dependents increases, and it is difficult for female doctors to work in hospitals for a long period of time until they take a responsible position.*** *These tendencies were not only typically shown in our hospital but in the entire Japanese health care system. ~~In other words, such differences might be related to delayed systems of childcare in Japan.~~* ***This is because Japanese society does not have a sufficient environment for women to continue their careers. Actually, according to the Global Gender Gap Report 2021[11] , Japan ranks 120th out of 156 countries in the comprehensive index of gender equality of health, education, economy, and politics,*** *indicating that the social progress of females in this country lags far behind other nations.*

Thank you for your especially important point. I corrected as you suggested.

11. ***Conclusions :*** *Our results show that a significantly higher proportion of physician-mothers were caught in a dilemma facing an increased home burden and clinical duties in the　hospital than physician-fathers during the pandemic. ~~This suggests that we have to redress this inequality as soon as possible to avoid such problems in the event of another such social crisis, which could occur at any time.~~* ***In order for female doctors to continue their careers and take responsible positions in the same way as male doctors, it is necessary to improve the delay in gender equality in Japanese society.***

We revised as follows (same as above No.2)

As we showed, female doctors could have not continued their careers and take responsible positions in the same way as male doctors. This is a social risk in the timing of a crisis, such as a pandemic.

12. *References*

Thank you for indicating areas for improvement. I have changed it as you suggested. I have also replaced the references No.5.
